# Lycium barbarum glycopeptide alleviates neuroinflammation in spinal cord injury via modulating docosahexaenoic acid to inhibiting MAPKs/NF-kB and pyroptosis pathways

**DOI:** 10.1186/s12967-023-04648-9

**Published:** 2023-10-31

**Authors:** Zhanfeng Jiang, Zhong Zeng, He He, Mei Li, Yuanxiang Lan, Jianwen Hui, Pengfei Bie, Yanjun Chen, Hao Liu, Heng Fan, Hechun Xia

**Affiliations:** 1 School of Clinical Medicine, Ningxia Medical University, Yinchuan, 750004 Ningxia Hui Autonomous Region People’s Republic of China; 2 Ningxia Key Laboratory of Stem Cell and Regenerative Medicine, Institute of Medical Sciences, General Hospital of Ningxia Medical University, Yinchuan, 750004 Ningxia Hui Autonomous Region China; 3 Department of Neurosurgery, General Hospital of Ningxia Medical University, Yinchuan, 750004 Ningxia Hui Autonomous Region People’s Republic of China

**Keywords:** Lycium barbarum glycopeptide, Docosahexaenoic acid, Neuroinflammation, Spinal cord injury

## Abstract

**Background:**

Lycium barbarum polysaccharide (LBP) is an active ingredient extracted from Lycium barbarum that inhibits neuroinflammation, and Lycium barbarum glycopeptide (LbGp) is a glycoprotein with immunological activity that was purified and isolated from LBP. Previous studies have shown that LbGp can regulate the immune microenvironment, but its specific mechanism of action remains unclear.

**Aims:**

In this study, we aimed to explore the mechanism of action of LbGp in the treatment of spinal cord injury through metabolomics and molecular experiments.

**Methods:**

SD male rats were randomly assigned to three experimental groups, and after establishing the spinal cord hemisection model, LbGp was administered orally. Spinal cord tissue was sampled on the seventh day after surgery for molecular and metabolomic experiments. In vitro, LbGp was administered to mimic the inflammatory microenvironment by activating microglia, and its mechanism of action in suppressing neuroinflammation was further elaborated using metabolomics and molecular biology techniques such as western blotting and q-PCR.

**Results:**

In vivo and in vitro experiments found that LbGp can improve the inflammatory microenvironment by inhibiting the NF-kB and pyroptosis pathways. Furthermore, LbGp induced the secretion of docosahexaenoic acid (DHA) by microglia, and DHA inhibited neuroinflammation through the MAPK/NF-κB and pyroptosis pathways.

**Conclusions:**

In summary, we hypothesize that LbGp improves the inflammatory microenvironment by regulating the secretion of DHA by microglia and thereby inhibiting the MAPK/NF-κB and pyroptosis pathways and promoting nerve repair and motor function recovery. This study provides a new direction for the treatment of spinal cord injury and elucidates the potential mechanism of action of LbGp.

**Supplementary Information:**

The online version contains supplementary material available at 10.1186/s12967-023-04648-9.

## Introduction

Spinal cord injury (SCI) is a highly disabling and lethal disease of the central nervous system (CNS) for which there are no effective treatment strategies. The incidence of SCI in China has increased in recent years due to various factors, such as tumors, hemorrhage, inflammation, and trauma [1]. Approximately 17,000 new patients with SCIs are reported annually across the United States [2].

Microglia are immune cells in the CNS that play a critical role in neuroinflammation, homeostasis, and stress in vivo [3]. Cytokine production mediated by overactivated microglia after SCI triggers an extensive inflammatory cascade; and during this process, substantial production of inflammatory factors and reactive oxygen species (ROS) exacerbates the secondary injury, chemokines recruits peripheral immune cells into the injured area, and reactive nitric oxide species, damage-related molecular patterns (DAMP), and inflammatory signals induce their entry into the tissues surrounding the injured area, which contributes to the clearance of pathogens and cellular debris [4–6].

Activation of the cytoplasmic inflammasome is a fundamental step in neuroinflammatory processes and a key trigger for neuronal pyroptosis [7]. To date, five receptor proteins have been confirmed to assemble into inflammasomes: the nucleotide-binding oligomerization domain (NOD) and leucine-rich repeat (LRR)-containing protein (NLR) family members NLRP1, NLRP3, and NLRP4 and the proteins absent in melanoma2 (AIM2) and pyrin [8]. SCI reportedly triggers NLRP3 inflammasome activation in spinal cord microglia [9]. The NOD-like receptor protein-3 (NLRP3) inflammasome, which is assembled with NLRP3, an apoptosis-associated speck-like protein containing a caspase recruitment domain (ASC), an endogenous “danger signal” followed by cysteine protease-1, and exogenous infection, is an important cytosolic protein complex [10]. The activation of the NLRP3 inflammasome requires two key steps and an activation step, in which the binding of DAMPs and pathogen-associated molecular patterns to pattern recognition receptors activates the Toll-like-receptor (TLR)-myeloid differentiation primary response protein MyD88-nuclear factor-kB (NF-kB) pathway to induce inflammasome transcription and trigger posttranslational modification. The second step is the assembly and activation of the inflammasome, and inflammasome activation can be achieved through three mechanisms, namely, ROS activation by NADPH oxidase, lipopolysaccharide (LPS) and ATP production, lysosomal rupture, and ion channel gating, and NADPH oxidase-mediated ROS play a vital role in this process [7, 11–13]. Activated NLRP3 nucleates pyroptosis-associated apoptotic protein-containing caspase activation and recruitment domain (CARD) helical fibrils to form ASC patches, which induce and activate pro-caspase-1 cleavage and promote the activation of pro-interleukin (IL)-1β, pro-IL-18, and GSDMD [11]. Cleavage of the N-terminal end of GSDMD drives pyroptosis, which leads to cell death and allows cells to release mature IL-1β and IL-18 [8, 14].

Lycium barbarum glycopeptide (LbGp) is a glycoprotein with immunological activity that was further purified and isolated from Lycium barbarum polysaccharide (LBP) [15]. Through metabolomic analysis (Ningxia Tianren GoJi Biotechnology, Ningxia, China), we previously revealed that LbGp comprises 368 metabolites, including 42 classified as other compounds (40%), 118 terpenoids (32%), 38 alkaloids (10%), 24 phenylpropanoids (6.5%), 17 phenols (4.6%), 17 flavonoids (4.6%), 4 amino acids (1.1%), 3 aliphatic acyls (0.8%), and 3 fatty acids (0.8%). However, the precise LbGp component that improves the inflammatory microenvironment in vivo by inhibiting the MAPK-NF-κB and pyroptosis-related pathways needs to be identified.

In the present study, we aimed to demonstrate that LbGp induces microglia to secrete DHA in vivo and that this secretion promotes nerve regeneration and motor function repair and improves the inflammatory microenvironment by inhibiting MAPK-NF-κB and pyroptosis-related pathways. These findings provide a new direction for the treatment of SCI. Accordingly, we performed in vivo and in vitro molecular and metabolite analyses.

## Methods and materials

### Animals

In total, 70 (8-week-old) male Sprague‒Dawley rats (300–350 g) were purchased from the Laboratory Animal Center of Ningxia Medical University. After surgery, all the rats were housed under continuous temperature (21 ± 3 °C) and humidity (50% ± 5%) conditions with a 12 h light/dark cycle. The experimental protocol was approved by the Laboratory Animal Ethical and Welfare Committee of the Laboratory Animal Center of Ningxia Medical University.

### Rat SCI model and treatment groups

The twelfth thoracic vertebral body (T12) hemisection model was established as previously described [16]. To establish the SCI model, rats were anesthetized with isoflurane (2–4% induction, 1.5% maintenance), and their body temperature was maintained at 35 ± 1 °C using a mini heating pad. The skin of the upper thoracic area was exposed after shaving and cleaning with betadine solution. To expose the T12, the fascia and muscle were bluntly dissected to avoid spinal dural injury during the incision of the dorsal lamina. A needle (26G) was bent 90° to establish a lateral hemisection, and the spinal cord was punctured dorsoventrally at the midline by inserting the needle 5 mm while avoiding damage to the dorsal spinal artery. The left half of the spinal cord was pulled and cut, and this step was repeated three times to ensure completeness of the spinal cord hemisection (Additional file 1: Fig. S2A, B). For pain management, carprofen (4 mg/kg; Rimadyl®, Zoetis, Florham Park, NJ, USA) and buprenorphine (0.2 mg/mL; Temgesic®, Intervet International, Boxmeer, the Netherlands) were administered continuously before surgery and every 8 h for three successive days after SCI.

Forty-five adult male rats were randomly assigned to three groups (15 rats per group): sham group, SCI group and post-SCI LbGp treatment group. LbGp was dissolved in 0.01 M phosphate-buffered saline at room temperature, and the solution was administered to the rats in the post-SCI LbGp treatment group via a nasogastric tube (50 mg/kg) once daily until day 28 post-SCI. The rats in the sham and SCI groups were administered 0.01 M phosphate-buffered saline 24 h after SCI.

### Behavioral testing

We next evaluated the hind limb motor function of rats after SCI. Three rats were selected from each group, and the Basso-Beattie-Bresnahan (BBB) test was used to score the motor function of the hind limbs. The BBB test is divided into 22 grades, with 21 points indicating normal paralysis and 0 points indicating paralysis. The scoring was performed at 8 p.m. in a double-blind manner. Each rat was measured three times, and the average value was recorded. The more detail of BBB Score as previously describe: the rats were placed on a circular platform with a diameter of 2 m. The walking and limb activity scores of the hindlimbs were observed and recorded. In the first stage (0–7 points), the joint activity of the hindlimbs of the animals was scored. In the second stage (8–13 points), the gait and the coordination of the hindlimbs were scored. In the third stage (14–21 points), the fine movements of the claws were judged. The scores for the three stages together totalled 21 points. Each group was scored 1 day before surgery and 1, 3, 7, 14, and 28 days post-injury (dpi) [17].

### Western blot

The rat microglia (RM) cell line (BNCC 360237) was purchased from the BeNa culture collection (Henan, China). First, RM cells were seeded in six-well plates (1 × 10^–5^ cells/well), activated with LPS (1 µg/ml L3129 Sigma) and ATP (5 mM; Sigma) for 4 h, and then treated with LbGp or DHA for 24 h to establish the LbGp treatment group or DHA treatment group, respectively. In addition, microglia activated with ATP + LPS were incubated with the FADS2 enzyme inhibitor SC26196 for 12 h and then incubated with LbGp for 24 h to construct the ATP + LPS + SC26196 + LbGp treatment group. Spinal cord tissue was harvested on day 7 post-SCI. Tissue or cell lysates were prepared using the Keygen protein extraction kit (KGP250 Keygen BioTECH China), and the protein concentration was measured with the BCA protein assay kit (KGP902 Keygen BioTECH China). Equal amounts of protein were separated with 10 or 15% sodium dodecyl sulfate‒polyacrylamide gels, transferred to polyvinylidene fluoride membranes, and blocked with 5% skim milk. Subsequently, the membranes were probed with the following primary antibodies at 4 °C overnight: anti-brain-derived neurotrophic factor (BDNF) (1:10,000) (Cat. Number: ab108319; Abcam UK), anti-glia-derived neurotrophic factor (GDNF) (1:2000) (Cat. Number: A14639; ABclonal China), anti-P-p-38 (1:1000) (Cat. Number: 28796–1-AP; Proteintech, China), anti-p38 (1:1000) (Cat. Number: 14064–1-AP; Proteintech, China), anti-P-JNK (1:1000) (Cat. Number: 80024–1-RR; Proteintech, China), anti-JNK (1:1000) (Cat. Number: 24164–1-AP; Proteintech, China), anti-P-p65 (1:1000) (Cat. Number: ab76302; Abcam, Cambridge, UK), anti-p65 (1:20,000) (Cat. Number: 80979–1-RR; Proteintech, China), anti-NLRP3 (1:2000) (Cat. Number: 27458–1-AP; Proteintech, China), anti-ASC (1:3000) (Cat. Number: 10500–1-AP; Proteintech, China), anti-caspase-1 p45 (1:1000) (Cat. Number: ab179515; Abcam UK), anti-caspase-1 p20 (1:2000) (Cat. Number: bs-10442R; Bioss, China), anti-GSDMD and anti-GSDMD-N (1:1000) (Cat. Number: ab219800; Abcam, UK), anti-pro-IL-18 and anti-IL-18 (1:10,000) (Cat. Number: 10663–1-AP; Proteintech, China), anti-IL-1β (1:1000) (Cat. Number: ab254360; Abcam, UK), and anti-β-actin (1:40,000) (Cat. Number: 81115–1-RR; Proteintech China). The membrane was washed with TBST, incubated with secondary antibody (1:20,000) (Cat. Number: ab6721 Abcam UK) for 1 h at room temperature, washed again, visualized with ECL reagent (SW134-01 Seven Biotech, China), and quantitatively analyzed using ImageJ software (National Institutes of Health, Bethesda, MD, USA).

### Cell viability assay

The Cell Counting Kit-8 (CCK-8) assay (Dojindo Laboratories Lot. CK04 Japan) was used to assess the toxicity of LbGp toward microglia. Microglia were plated in 96-well plates at 7 × 10^–3^ cells/well, and different concentrations of LbGp were added (100, 200, and 400 µg/ml). The microglia were then incubated at 37 °C in a 5% CO_2_ incubator for 24 h. The supernatant was discarded, and 100 µl of medium containing 10% CCK-8 reagent was added to each well. The plate was then incubated at 37 °C in a 5% CO_2_ incubator for 2 h in the dark. The absorbance of each well was detected with microplate reader at a wavelength of 450 nm.

### Enzyme-linked immunosorbent assay analysis (ELISA)

The level of DHA in each group was detected by COIBO BIO (Shanghai, China). RM cells were seeded in six-well plates (1 × 10^–5^ cells/well), activated with LPS (1 µg/ml; L3129, Sigma) and ATP (5 mM; Sigma) for 4 h, and treated with LbGp for 24 h. The cells were centrifuged at 4 °C and 800*g* for 10 min, and 200 µl of supernatant was then collected for DHA detection. DHA assays were performed according to the protocol provided by the COIBO BIO (CB14901-Ra, Shanghai, China).

### RNA preparation and reverse transcription-quantitative PCR

Microglia were counted and plated into a six-well plate at a density of 1 × 10^–5^ cells/well. Upon reaching 80% confluency, the cells were incubated with 1 µg/ml LPS for 12 h and then treated with different concentrations of LbGp (100, 200, and 400 µg/ml) for 24 h. After three washes with cold phosphate-buffered saline (PBS), 1 ml of TRIzol was added, and the mixture was pipetted into a 1.5-ml EP tube. After the addition of 200 µl of chloroform, the prepared samples were centrifuged at 9000*g* at 4 °C for 15 min. The supernatant was aspirated, and an equal volume of isopropanol was added. The mixture was then centrifuged at 9000*g* at 4 °C for 15 min. The supernatant was removed, and 75% ethanol was added. After two rounds of centrifugation at 6000*g* for 5 min at 4 °C, double-distilled water (ddH2O) was added, and the RNA concentration was measured. The reverse transcription kit and real-time quantitative PCR kit were purchased from Vazyme (lot. R302-01, Q411-02; Nan Jing, China), and the assays were performed according to the manufacturer’s instructions. The gene-specific sequences of the primers used are shown in Additional file 2: Table S1. Relative gene expression was measured using the 2^−^ ^△△ct^ method.

### Immunofluorescence staining

Rat spinal cord tissue was collected on day 7 post-SCI and subjected to fixation, paraffinization, dewaxing, dehydration, antigen extraction, and blocking according to the standard protocol. Subsequently, tissue sections were incubated with the following primary antibodies for 24 h: anti-NLRP3, anti-ASC, anti-caspase-1, and anti-NeuN (1:2000) (Cat. Number: 6975-1-AP; Proteintech, China). Next, tissue sections were incubated with fluorescent-labeled secondary antibody colored green (1:500) (ab150077; Abcam UK), red (1:200 SA00013-4; Proteintech, Wuhan, China), rose red (1:200 GB21303 Servicebio China), pink (1:200 GB1232 Servicebio China) for 3 h, and the nuclei were stained with DAPI. Images were scanned using an ortho-fluorescence microscope (Nikon Eclipse C1) and a fluorescence microscope (Olympus, Japan). Quantitative analysis was performed using ImageJ.

### Metabolite processing and identification

One week post-SCI, the rats in the three groups (A: sham group, B: SCI group, C: LbGp treatment groups; n = six rats/group) were anesthetized under excessive isoflurane, and the dorsal skin surface was incised. The vertebrae were cut, and the injured area of the spinal cord tissue was harvested (length of approximately 50 mm in length, weight of approximately 30 mg). The harvested spinal tissue was rapidly placed in liquid nitrogen and transferred to a − 80 °C refrigerator for metabolomic sequencing analysis.

Metabolomics was performed by Shanghai Luming Biotech Co., Ltd. All reagents were of high-performance liquid chromatography (HPLC) grade. L-2-chlorophenylalanine was purchased from Shanghai Heng Chuang Biotechnology (Shanghai, China), and methoxyamine hydrochloride (97%), pyridine, n-hexane, and BSTFA with 1% TMCS were purchased from CNW Technologies GmbH (Düsseldorf, Germany). Chloroform was obtained from Titan Chemical Reagent (Shanghai, China), and water and methanol were obtained from Thermo Fisher Scientific (Waltham, MA, USA).

Briefly, 30 mg of sample was added to a 1.5-ml centrifuge tube, and after the addition of two small steel beads and 600 µl of methanol–water (V:V + 4:1, containing L-2-chlorophenylalanine, 4 µg/ml), the tube was incubated for 2 min. The samples were placed in a grinder (60 Hz, 2 min), and after the addition of 120 µl of chloroform, the samples were vortexed for 2 min. Ultrasonic extraction was performed in an ice-water bath for 10 min at − 40 °C or for 30 min. Subsequently, centrifugation was performed for 10 min (12,000*g* 4 °C), and 150 µl of the supernatant was placed in a glass derivatization bottle. The sample was then dried using a centrifugal concentrator desiccator. Subsequently, 80 µl of methoxyamine hydrochloride pyridine solution (15 mg/ml) was added to the glass derivatized vial, and the sample was vortexed for 2 min and placed in an incubator at 37 °C for 60 min to perform the oxidation reaction. After removing the sample, 50 µl of BSTFA derivatization reagent and 20 µl of n-hexane were added, and 10 internal standards (c8/c9/c10/c12/c14/c16/c18/c20/c22/c24, all prepared in chloroform; 10 µl) were added. The mixture was vortexed for 2 min and reacted at 70 °C for 60 min. The obtained samples were placed at room temperature for 30 min for gas chromatography‒mass spectrometry (GC‒MS) metabolomic analysis. Quality control samples were prepared by mixing equal volumes of extracts from all the samples.

GC‒MS was performed using a Db-5MS capillary column (30 × 0.25 mm × 0.25 µm; Agilent J&W Folsom, CA, USA), high-purity helium (purity ≥ 99.999%) as the carrier gas, a flow rate of 1.0 ml/min, and an injection port temperature of 260 °C. The injection volume was 1 µl, and the injection was split with a solvent delay of 5 min. The program temperatures were as follows: the temperature of the column oven was initially set to 60 °C and maintained at 60 °C for 0.5 min; the program temperature was then increased to 125 °C at 8 °C/min, to 210 °C at 8 °C/min, to 270 °C at 15 °C/min and to 305 °C at 20 °C/min and maintained at 305 °C for 5 min.

The electron bombardment ion source had the following parameters: ion source temperature, 230 °C; quadrupole temperature, 150 °C; and electron energy, 70 eV. The scanning mode was full scan mode (SCAN) with a mass scanning range of 50–500 m/z.

The collected GS/MS raw data were converted into abf format using Analysis Base File Convert for rapid data retrieval. The data were then imported into MS-DIAL software for characterization, MS2Dec deconvolution, peak alignment, peak identification, peak detection, wave filtering, and missing value interpolation. Metabolites were characterized based on the LUG database. The data matrix included the signal intensity, retention index, retention time, mass-to-charge ratio, sample information, and peak name for each species. After screening, all peak signal intensities were segmented and normalized for each sample according to an internal criterion of RSD ˃ 0.3. After data normalization, redundancy removal and peak merging were performed to obtain a data matrix.

The matrix was imported into R for principal component analysis to observe the overall distribution between samples and the stability of the entire analysis process. The differential metabolites between groups were identified by using orthogonal partial least squares discriminant analysis (PLS-DA). The model quality was assessed by sevenfold cross-validation and a 200-response permutation test.

The projected importance values of the variables in the OPLS-DA model were used to rank the overall contribution of each variable to the group discrimination. Two-tailed Student’s t test was used to verify whether the difference in metabolites between groups was significant. Differential metabolites that met the following criteria were selected: variable importance of projection value ˃ 1.0 and p value < 0.05.

#### Statistical analysis

The data are presented as the means ± S.Ds. One-way analysis of variance (ANOVA) was used to assess the differences identified from comparisons of multiple groups. All statistical analyses were performed using GraphPad Prism (version 9.3). p < 0.05 was considered to indicate statistical significance.

## Result

### LbGp promotes motor function recovery after SCI.

**Fig. 1:**
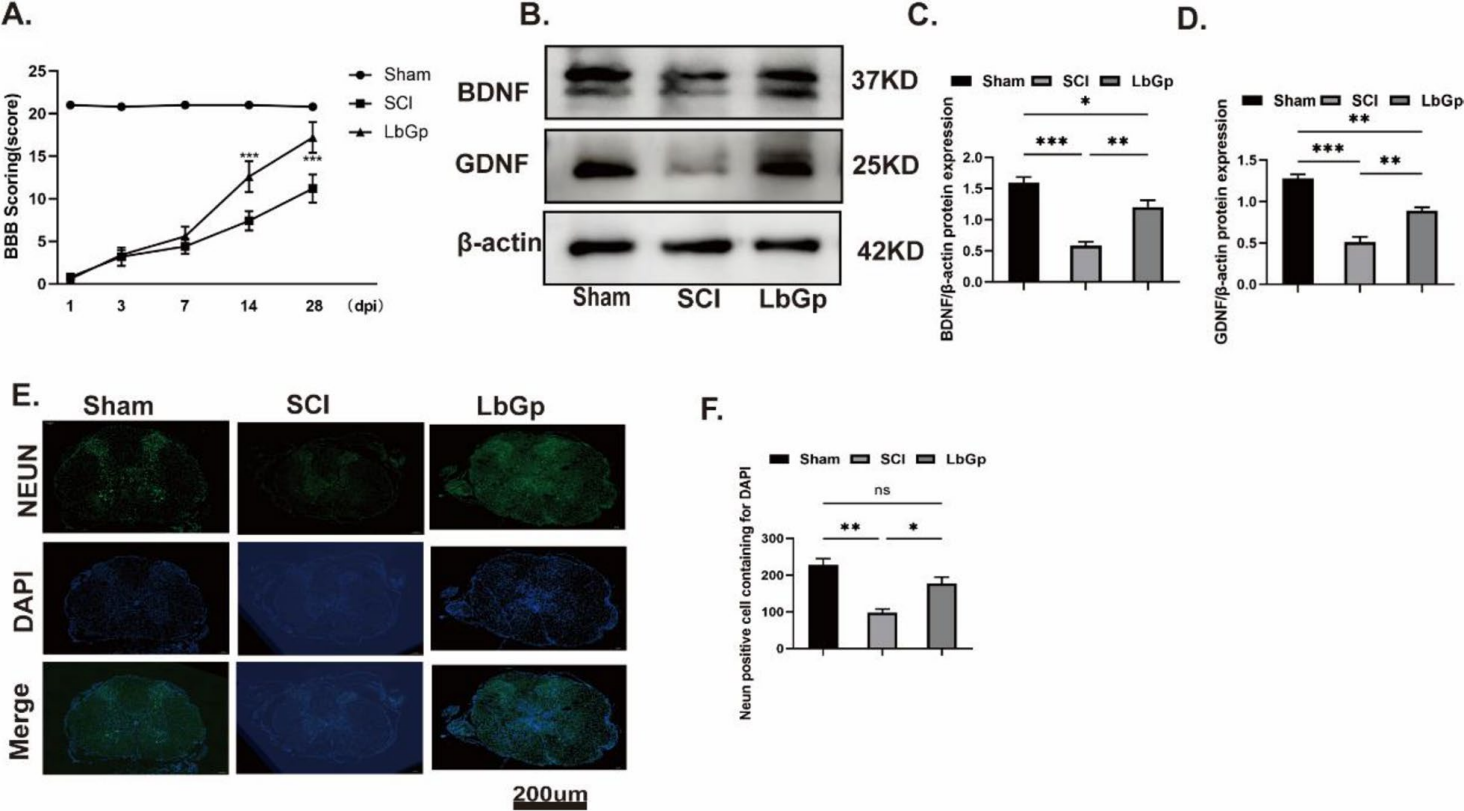
*Lycium* barbarum glycopeptide promotes nerve regeneration and benefits motor function recovery. **A** The motor function of each group was evaluated based on the BBB score. The score of the LbGp treatment group was higher than that of the SCI group after the second week and did not significantly differ from that of the sham group. n = 5 for each group;***p < 0.001, SCI vs. LbGp treatment group. **B**–**D** The expression levels of BDNF and GDNF in spinal cord tissue were detected by western blotting. **E**, **F** Fluorescence staining micrographs of NeuN protein in spinal cord tissues of the different groups. The data are presented as the means ± SEMs of at least 3 independent experiments, Scale bar, 200 µm; n = 3 per group;*p < 0.05, **p < 0.01, ***p < 0.001, and ****p < 0.0001 versus each group

LbGp was purified from LBP and contains 368 metabolites (Additional file 1: Fig. S1A). LBP is known to induce nerve regeneration and promote motor function recovery [18]. To test our conjecture, we established a rat spinal cord hemisection model, orally administered LbGp after the operation, and assessed the motor function of the different treatment groups up to 28 days after surgery using the BBB motor function score. Based on the BBB test results, the score of the SCI group was significantly lower than that of the sham group, and the score of the LbGp group did not significantly differ from that of the SCI group during the first week post-SCI but was higher than that of the SCI group during the second week (Fig. 1A). These results revealed that LbGp treatment could improve the motor function of rats. On day 14, the BBB motor function score of the LbGp intervention group was higher than that of the SCI group.

### LbGp regulates neurotrophic factors to promote nerve regeneration

Previous studies have shown that BDNF and GDNF play an important role in promoting nerve regeneration [19]. To further elucidate the potential role of LbGp in nerve regeneration, we detected the expression levels of BDNF and GDNF in SCI by western blotting and that of NeuN protein by immunofluorescence. Because the inflammatory microenvironment was altered prior to behavioral changes in the animals, we further investigated the mechanism of motor function recovery in rats by examining the levels of brain-derived neurotrophic factor (BDNF) and glia-derived neurotrophic factor (GDNF) in the spinal cord tissues of rats on day 7. On day 7, the protein expression levels of BDNF and GDNF were significantly higher in LbGp-treated rat spinal cord tissues than in the tissues of the SCI group, as determined by western blotting (Fig. 1B–D; Additional file 1: Fig. S3A, B). On day 7, the expression of NeuN protein in spinal cord tissue was consistent with the above-described results, as determined by tissue immunofluorescence experiments (Fig. 1E, F). Therefore, we hypothesize that LbGp could promote neuronal repair and improve the motor function of rats after SCI by modulating BDNF and GDNF expression.

### LbGp inhibits NF-kB and pyroptosis-related proteins in vivo

**Fig. 2:**
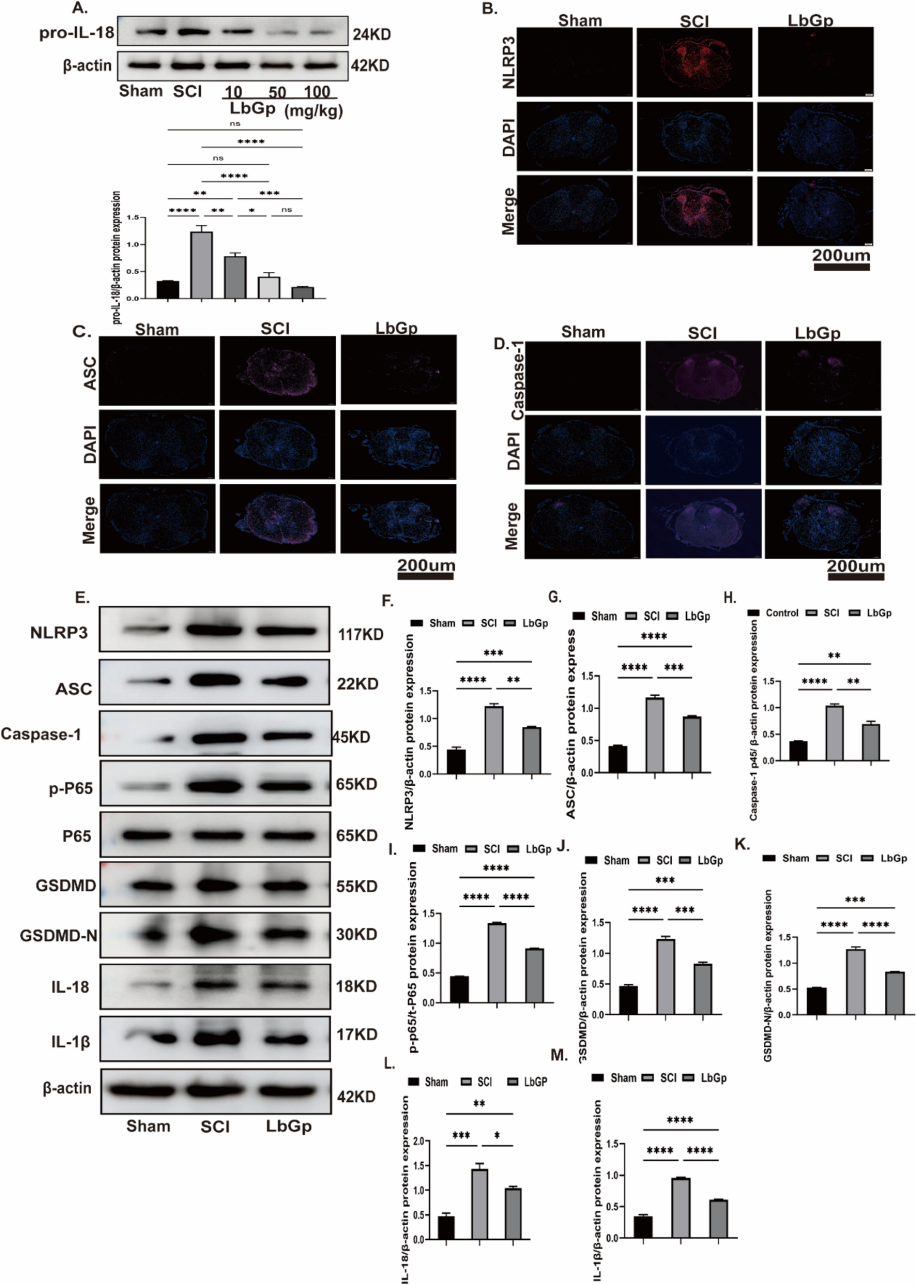
*Lycium* barbarum glycopeptide inhibits NF-kB-and pyroptosis-related proteins in vivo. **A** SCI treatment with different doses (10 mg/kg-100 mg/kg) of LbGp reduced the pro-IL-18 protein expression levels. **B**–**D** Fluorescence staining micrographs of NLRP3, ASC,andcaspase-1 protein on the 7th day in spinal cord tissues of the different groups. **E**–**M** On day 7, the protein expression levels of NLRP3, ASC, caspase-1, P-p65, P-65, GSDMD, GSDMD-N, IL-18, and IL-1β in spinal cord tissue from each group were detected by western blotting. The data are presented as the means ± SEMs of at least 3 independent experiments. Scale bar, 200 µm; n = 3 per group;*p < 0.05,**p < 0.01, ***p < 0.001, and ****p < 0.0001 versus each group

The role of LbGp in inhibiting neuroinflammation has been demonstrated [20]. To further confirm the role of LbGp in neuroinflammation, we administered different concentrations of LbGp after SCI by gavage and identified the optimal concentration based on the therapeutic effect. The inhibitory effect of different concentrations of LbGp on neuroinflammation was assessed by western blotting. Rats were treated with different concentrations (10, 50, and 100 mg/kg) of LbGp after SCI, and the inflammatory factor pro-IL-18 in the different treatment groups was detected. We found that LbGp induced superior inhibition of the inflammatory factory Pro-IL-18 at 50 mg/kg (Fig. 2A) (Additional file 1: Fig. S3C). The accumulated body of evidence shows that LBP can inhibit the expression of the NLRP3 inflammasome and ROS production to suppress inflammation [21]. To further elucidate the mechanism of the role of LbGp in SCI-induced neuroinflammation, we detected the levels of NLRP3, ASC, and caspase-1 by immunofluorescence and western blotting. The expression levels of NLRP3, ASC, and caspase-1 proteins determined by tissue immunofluorescence were significantly lower in the LbGp group than in the SCI group (Fig. 2B–D). Based on the western blot analysis, the rats in the SCI group exhibited elevated expression of P-p-65, NLRP3, ASC, caspase-1p45, GSDMD, GSDMD-N, IL-1β and IL-18 proteins in the spinal cord on day 7 post-SCI, and these expression levels were significantly reduced in the LbGp group (Fig. 2E–M) (Additional file 1: Fig. S3D, E). Taken together, these data indicate that the oral administration of LbGp after SCI can suppress neuroinflammation.

### LbGp inhibits MAPKs/NF-kB and pyroptosis-related proteins in vitro

**Fig. 3:**
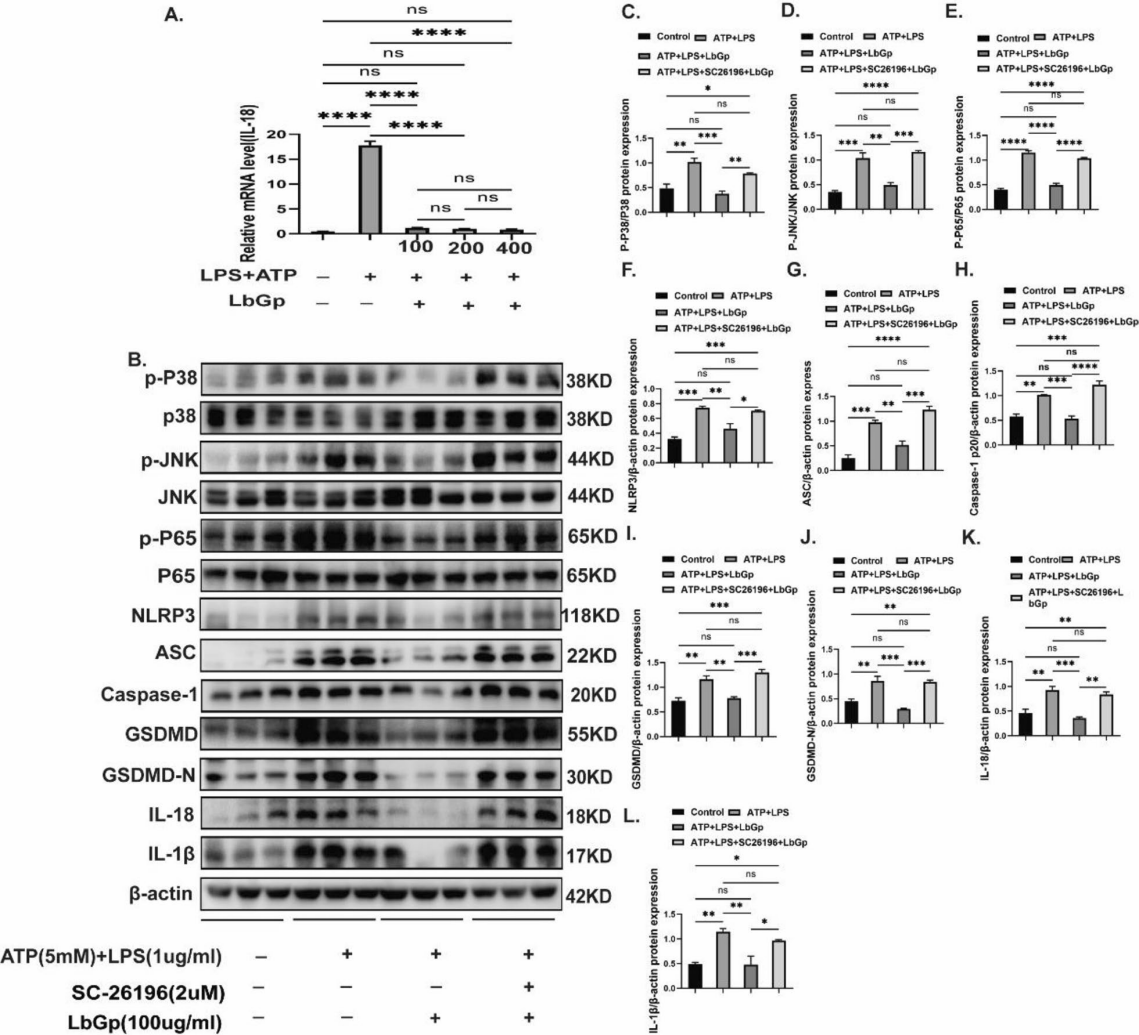
*Lycium barbarum *glycopeptide inhibits microglial MAPKs/NF-kB and pyroptosis-related pathways. **A** mRNA expression level of IL-18 in ATP + LPS-stimulated microglia treated with different doses(100 µg/kg–400 µg/ml) of LbGp. **B**–**L** The expression levels of P-p38, P38, p-JNK, JNK, P-p65, P-65, NLRP3, caspase-1-p20, ASC, GSDMD, GSDMD-N, IL-18, and IL-1β protein in each group of activated microglia were detected by western blotting. The data are presented as the means ± SEMs of at least 3 independent experiments, *p < 0.05, **p < 0.01, ***p < 0.001, and ****p < 0.0001 versus each group

As mentioned above, LbGp can inhibit the NF-kB and pyroptosis pathways to suppress neuroinflammation in vivo, but the target cells on which it acts are unclear. Microglia are intrinsic immune cells in the CNS that are involved in the immune response in vivo and play an essential role in neuroinflammation [22]. To determine the effect of LbGp on microglia, cell viability was examined by a CCK-8 assay. The results indicated no significant changes in cell viability after incubation with 100–400 µg/ml LbGp (Additional file 1: Fig. S4A). To further assess the optimal drug concentration for the LbGp-mediated inhibition of inflammation triggered by microglia, we incubated activated microglia with LbGp at different concentrations (100, 200 and 400 µg/ml) for 24 h and assayed the mRNA expression levels of the inflammatory factor IL-18. The RNA level of IL-18 was reduced in the LbGp groups (100, 200 and 400 µg/ml), and no significant difference was found among the LbGp groups (Fig. 3A). Based on the abovementioned results, we administered 100 µg/ml LbGp after microglial activation. As previously described, the administration of LbGp treatment after the activation of microglia resulted in lower expression of P-p38, P-JNK, P-p65, NLRP3, ASC, caspase-1p20, GSDMD, GSDMD-N, IL-18, and IL-1β proteins in the LbGp-treated group compared with the ATP + LPS group (Fig. 3B–K). Similarly, in vitro experiments showed that microglial activation followed by LbGp intervention can suppress neuroinflammation.

### LbGp inhibits MAPKs/NF-kB and pyroptosis-related proteins by modulating DHA

**Fig. 4:**
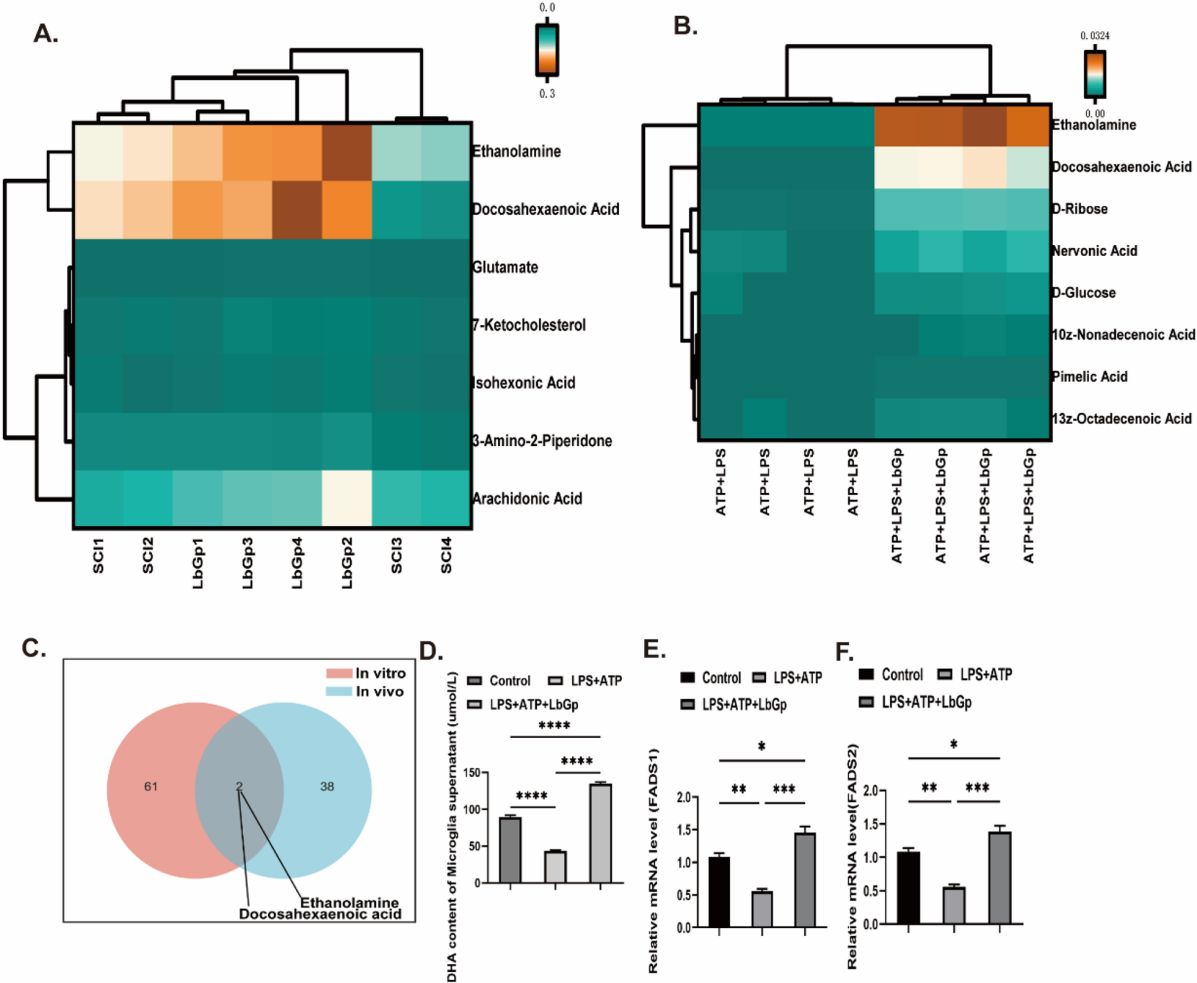
*Lycium* barbarum glycopeptide induces microglia to secrete DHA. **A** Heat map of differential metabolic substances between the SCI and LbGp groups in spinal cord tissue(In vivo). **B** Heat map of differential metabolic substances between the ATP + LPS and LbGp treatment groups in microglial supernatants (In vitro). **C** Venn diagram of elevated metabolites in spinal cord tissues from the LbGp-treated group and microglia from the LbGp-treated group. **D** Microglia were activated with ATP + LPS for 4 hand incubated with LbGp for 24 h, and the DHA levels in the cell supernatants were assayed. **E**, **F** mRNA expression levels of FADS1 and FADS2 in ATP + LPS-stimulated microglia treated with LbGp. The data are presented as the means ± SEMs of at least 3 independent experiments; n = 6per group;*p < 0.05, **p < 0.01, ***p < 0.001, and ****p < 0.0001 versus each group

As described previously, LbGp inhibited neuroinflammation both in vivo and in vitro, and to further clarify the mechanism of LbGp treatment for SCI by metabolomic analysis, 66 and 38 metabolites were found to be elevated in cell supernatants and tissues, respectively, in the LbGp-treated group (Additional file 1: Fig. S5A–E). Pairwise comparisons revealed that both ethanolamine and DHA were increased in tissue and cell supernatants after LbGp intervention (Fig. 4C). At present, the role of ethanolamine in neuroinflammation is unclear [23–25]. The view that DHA suppresses inflammation by inhibiting the NIRP3 protein in activated macrophages has been well confirmed [26–28]. DHA intervention after SCI can inhibit neuroinflammation in rats [29]. Therefore, we hypothesized that LbGp may suppress neuroinflammation by regulating DHA production by microglia. To test this hypothesis, we administered SC-26196, an inhibitor of FADS2, a key enzyme for DHA production, before LbGp intervention, and the results showed that the inhibitory effect of LbGp on inflammation was significantly weakened after reduced DHA production (Fig. 3B–L). Collectively, our data indicate that LbGp regulates the production of DHA by microglia and thereby suppresses neuroinflammation.

### LbGp induces DHA secretion from microglia

To determine whether LbGp can induce DHA secretion from microglia, we analyzed cell supernatants by ELISA and found that the level of DHA was significantly elevated in the LbGp intervention group (Fig. 4D). Metabolomic analysis showed that microglia are rich in DHA, providing the possibility of DHA secretion [30]. FADS1 and FADS2 play an essential role in the process of DHA synthesis [31]. Q-PCR results showed that LbGp intervention significantly contributed to high mRNA expression of FADS1 and FADS2, key enzymes for DHA production (Fig. 4E, F). As mentioned previously, our data demonstrate that LbGp promotes high expression of the key enzymes FADS1 and FADS2 in microglia and thereby stimulates DHA secretion from microglia.

### DHA inhibits MAPKs/NF-kB and pyroptosis-related proteins in vitro

**Fig. 5:**
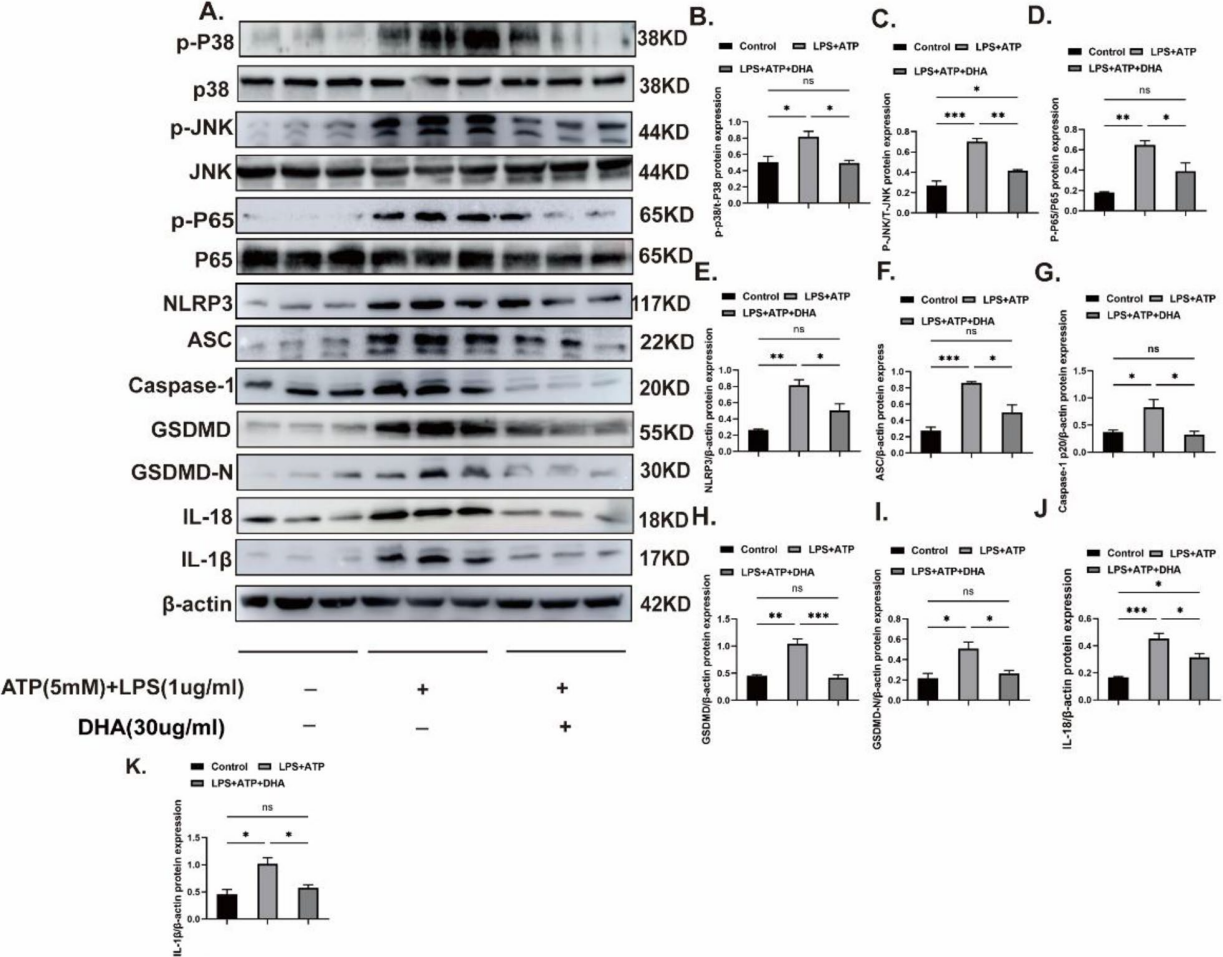
DHA inhibits microglial MAPKs/NF-kB and pyroptosis-related pathways. **A**‒**K** The expression levels of P-p38, P38, P-JNK,JNK, P-p65, P-65, NLRP3, caspase-1-p20, ASC, GSDMD, GSDMD-N, IL-18, and IL-1β protein in each group of activated microglia were detected by western blotting. The data are presented as the means ± SEMs of at least 3 independent experiments;*p < 0.05, **p < 0.01, ***p < 0.001, and ****p < 0.0001 versus each group

A previous study suggested that DHA can inhibit microglia-induced neuroinflammation [32]. Therefore, we further verified its role in the MAPK/NF-kB and pyroptosis pathways by Western blotting. As previously described, the administration of DHA after the activation of microglia resulted in lower expression of P-p38, P-JNK, P-p65, NLRP3, ASC, caspase-1p20, GSDMD, GSDMD-N, IL-18, and IL-1β proteins in the DHA-treated group (Fig. 5A–K). Our in vitro results validate that DHA has an inhibitory function on neuroinflammation.

## Discussion

**Fig. 6:**
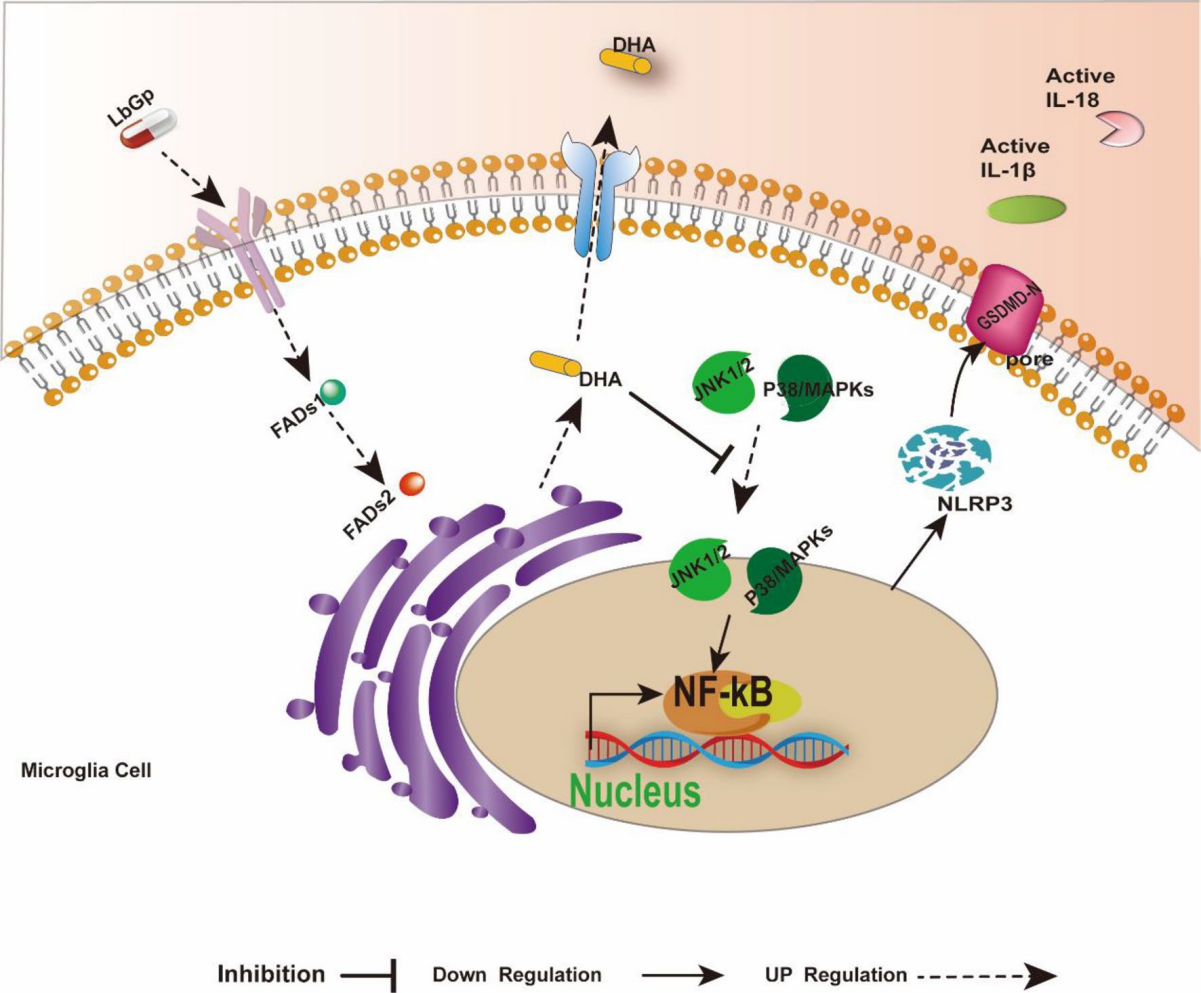
Model illustrating the role of LbGp in the treatment of SCI by inducing microglia to secrete DHA to inhibit the MAPK/NF-κB and pyroptosis pathways. Activation of microglia and release of inflammatory factors following SCI. LbGp stimulates microglia to produce DHA by regulating the key enzymes FADS1 and FADS2 in microglia, and thus, DHA can improve neuro inflammation by inhibiting the MAPK/NF-kB and pyroptosis pathways group.

This study provides the first demonstration that orally administered LbGp can improve the inflammatory microenvironment in SCI and promote spinal cord repair by inhibiting the MAPK-NF-κB/pyroptosis-related pathways, but the exact mechanism has not been previously reported. Based on previous reports and the results obtained in the present study, we illustrate the mechanism of Lycium barbarum glycopeptide treatment on the spinal cord injury (Fig. 6).

SCI is often accompanied by hemorrhage, peripheral tissue edema, secondary neuroinflammation, and glial scar formation, and these adverse factors severely limit nerve regeneration and functional recovery [33, 34]. SCI has two stages: primary SCI includes spinal cord tissue breakdown, hemorrhage, and destruction of the glial membrane [33]. Primary SCI is followed by delayed and progressive tissue injury. Ionic homeostasis imbalance in tissues after SCI leads to mitochondrial dysfunction and the release of reactive oxygen species, resulting in oxidative stress damage to tissue; damaged cells lead to the upregulation of excitatory amino acids and excitotoxicity, leading to apoptotic necrosis; inflammatory cells such as microglia and macrophages infiltrate the blood‒brain barrier, disrupt the damaged area and release inflammatory factors [35].

In recent years, an increasing number of herbal extracts have been used to study their neuroprotective mechanisms of action. It has been reported that Gastrodia elata Blume could increase the viability of embryonic neural progenitor cells under hypoxic conditions by improving their DNA damage repair ability [36]. Danshen extract plays a beneficial role in the recovery of locomotor function following SCI in rats. Moreover, this effect may be associated with the promotion of axonal regeneration, the upregulation of BDNF, and the activation of microglia [37]. Iridoid glycosides of Paederia scandens exert antinociceptive effects, which may be partly related to inhibition of the NO/cGMP/PKG signaling pathway in a rat spared nerve injury model of neuropathic pain [38]. Previous research has revealed that aloe-emodin protects against brain damage, and this effect is primarily attributed to the antioxidant and anti-neuroinflammatory properties of AE via PI3K/AKT/mTOR and NF-kB activation [39]. LBP is an active ingredient extracted from Lycium barbarum that can improve the inflammatory microenvironment [40, 41]. However, the mechanism of action of the LBP derivative LbGp in SCI is unclear.

After SCI, a large amount of ROS and ATP can activate the release of NLRP3, ASC, caspase-1, GSDMD and other key proteins related to pyroptosis and related inflammatory factors, which play a very important role in SCI [11, 42]. To further verify the mechanism of action of LbGp in inhibiting pyroptosis in SCI, we found that LbGp treatment could inhibit the expression of the key proteins P-p38/p-JNK in the MAPK pathway, P-p65 in the NF-kB pathway, NLRP3, ASC, caspase-1, GSDMD and downstream inflammatory factors such as IL-18 and IL-1β by western blotting. The specific mechanism of pyroptosis inhibition by LbGp treatment has not been clarified. Herein, the levels of the unsaturated fatty acid DHA were elevated in the LbGp groups, as determined by assessing the metabolism of microglia and tissues. Previous studies have shown that the unsaturated fatty acid DHA can inhibit NLRP3 and other key proteins of pyroptosis [26]. Unsaturated fatty acids reduce injury-related oxidative stress, decrease the responses of microglia/macrophages, and improve motor function and bladder function recovery in rats [43]. Following entry into the brain, DHA is esterified into and recycled among membrane phospholipids, contributing to the distribution of DHA in brain phospholipids. During neurotransmission and following brain injury, DHA is released from membrane phospholipids and converted to bioactive mediators that regulate signaling pathways important to synaptogenesis, cell survival, and neuroinflammation and may be relevant to the treatment of neurological diseases [44]. A previous study showed that 3 months of DHA treatment prevents microglial activation after ischemic injury, reduces the size of ischemic lesions, and increases the level of the antiapoptotic molecule Bcl-2 in the brain [45]. In addition, inhibition of the DHA-producing pathway in microglia reduced the effect of LbGp on improving neuroinflammation, which suggests that LbGp inhibits SCI pyroptosis by regulating the unsaturated fatty acid DHA. Accordingly, LbGp contains diverse metabolites (Additional file 1: Fig. S1A), but which metabolite plays the main role in inducing the release of DHA from microglia is unclear and needs to be further elaborated in future experiments.

LbGp can promote BDNF and GDNF to repair nerve damage. GDNF is a potent promoter of central and peripheral neurons, and BDNF is a major regulator of energy homeostasis that upregulates antioxidant enzymes to enhance the repair of damaged neurons, promote the differentiation of neurons and stem cells, promote neural protrusion growth and synapse formation, and prevent programmed cell death and apoptosis [19, 46–49]. DHA can promote high BDNF expression [50]. We found that LbGp promoted high expression of BDNF and GDNF in vivo, and Western blotting results further validated this conclusion. The results of an immunofluorescence analysis for the NeuN protein in spinal cord tissue indicated that the LbGp treatment promoted neuronal repair, and an analysis of the behavioral BBB function scores of rats showed that LbGp can improve the motor function of rats after SCI. The BBB motor function scores suggested that treatment with LbGp significantly improved the motor function of rats within 2 weeks after SCI. Therefore, we hypothesize that LbGp plays a role in the upregulation of BDNF and GDNF by modulating DHA to promote the recovery of motor function in rats.

## Conclusion

To our knowledge, we provide the first report of the mechanism of action of LbGp, a polysaccharide derivative of LBP, in the treatment of SCI, which involves the induction of microglia to secrete DHA to inhibit cellular pyroptosis. LbGp is widely available at low cost and has an excellent safety profile, and the findings will thus provide a new direction for the treatment of spinal cord injuries.

### Supplementary Information

**Additional file 1. Fig. S1.** Composition of Lycium barbarum glycopeptide. **Fig. S2**. Rat spinal cord hemisection model. **Fig. S3**. Effect of Lycium barbarum glycopeptide treatment on the spinal cord injury. **Fig. S4**. Cell viability of microglia treated with different concentration of Lycium barbarum glycopeptide. **Fig. S5**. Heatmap of differential metabolic substances between three groups in vivo and vitro.

**Additional file 2: Table S1.** Gene specific primer sequences of IL-18, FADS1 and FADS2.

## Data Availability

The datasets used and/or analyzed in this study are available from the corresponding author on reasonable request.

## Abbreviations

ASC: Apoptosis-associated speck-like protein
BBB: Basso-Beattie-Bresnahan
BDNF: Brain-derived neurotrophic factor
DAMP: Damage-related molecular patterns
DHA: Docosahexaenoic acid
GDNF: Glia-derived neurotrophic factor
GSDMD: Gasdermin D
LBP: Lycium barbarum polysaccharide
LbGp: Lycium barbarum glucopeptide
NLRP3: NOD-like receptor protein-3
NADPH: Nicotinamide adenine dinucleotide phosphate oxidase
ROS: Reactive oxygen species
SCI: Spinal cord injury
