# Supplementary material for: Lycium barbarum glycopeptide alleviates neuroinflammation in spinal cord injury via modulating docosahexaenoic acid to inhibiting MAPKs/NF-kB and pyroptosis pathways
Source: J Transl Med. 2023 Oct 31;21:770. doi: 10.1186/s12967-023-04648-9 (PMC10617163; doi:10.1186/s12967-023-04648-9)
Supplement: Supplementary file 1 — Additional file 1. Fig. S1. Composition of Lycium barbarum glycopeptide. Fig. S2. Rat spinal cord hemisection model. Fig. S3. Effect of Lycium barbarum glycopeptide treatment on the spinal cord injury. Fig. S4. Cell viability of microglia treated with different concentration of Lycium barbarum glycopeptide. Fig. S5. Heatmap of differential metabolic substances between three groups in vivo and vitro. [file 12967_2023_4648_MOESM1_ESM.docx]

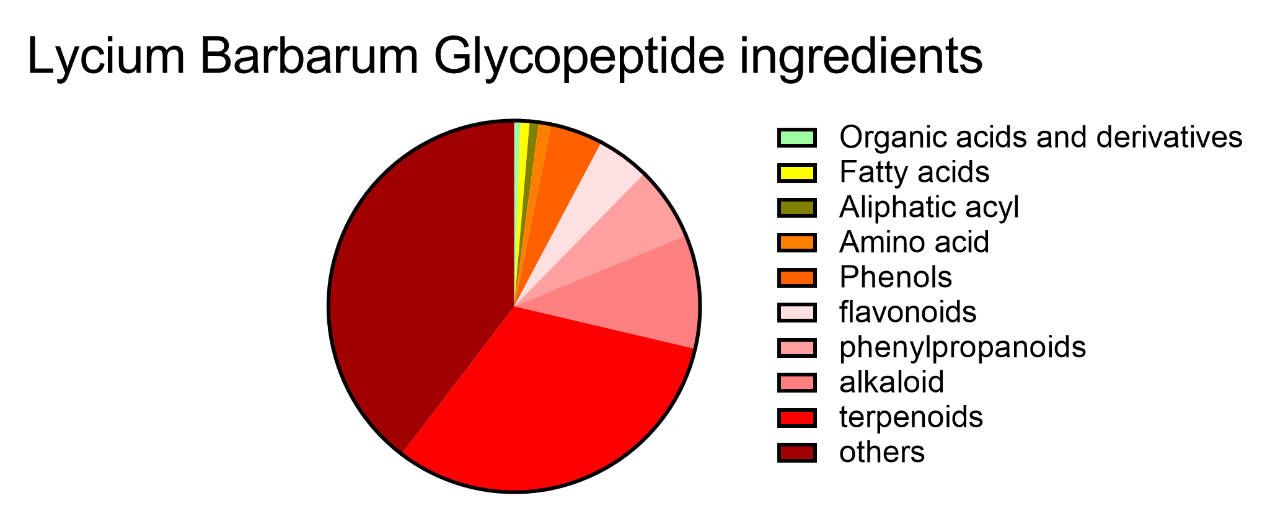


**Fig.S1 Composition of Lycium barbarum glycopeptide.** (A)Composition of Lycium barbarum glycopeptide;


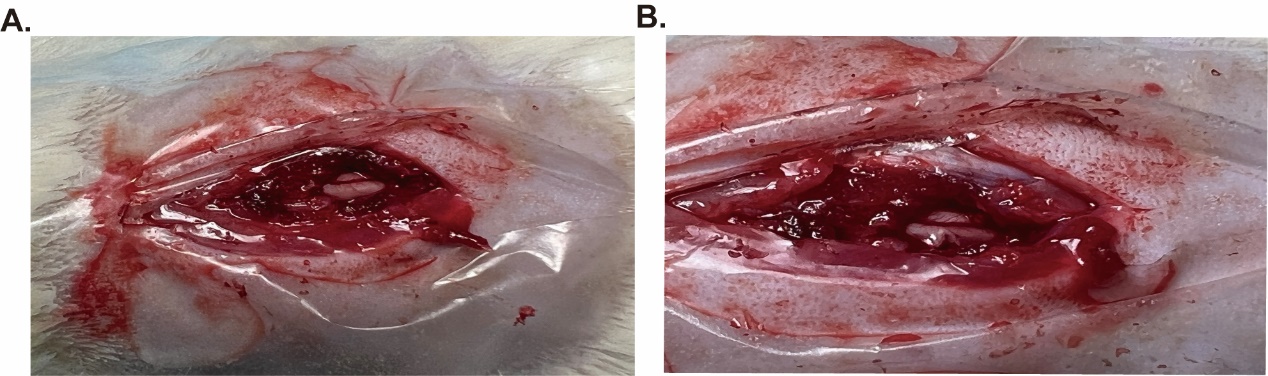


**Fig. S2 Rat spinal cord hemisection model.** (A). Establishment of rat spinal cord hemisection model(before)；（B）.Establishment of rat spinal cord hemisection model(after)

**
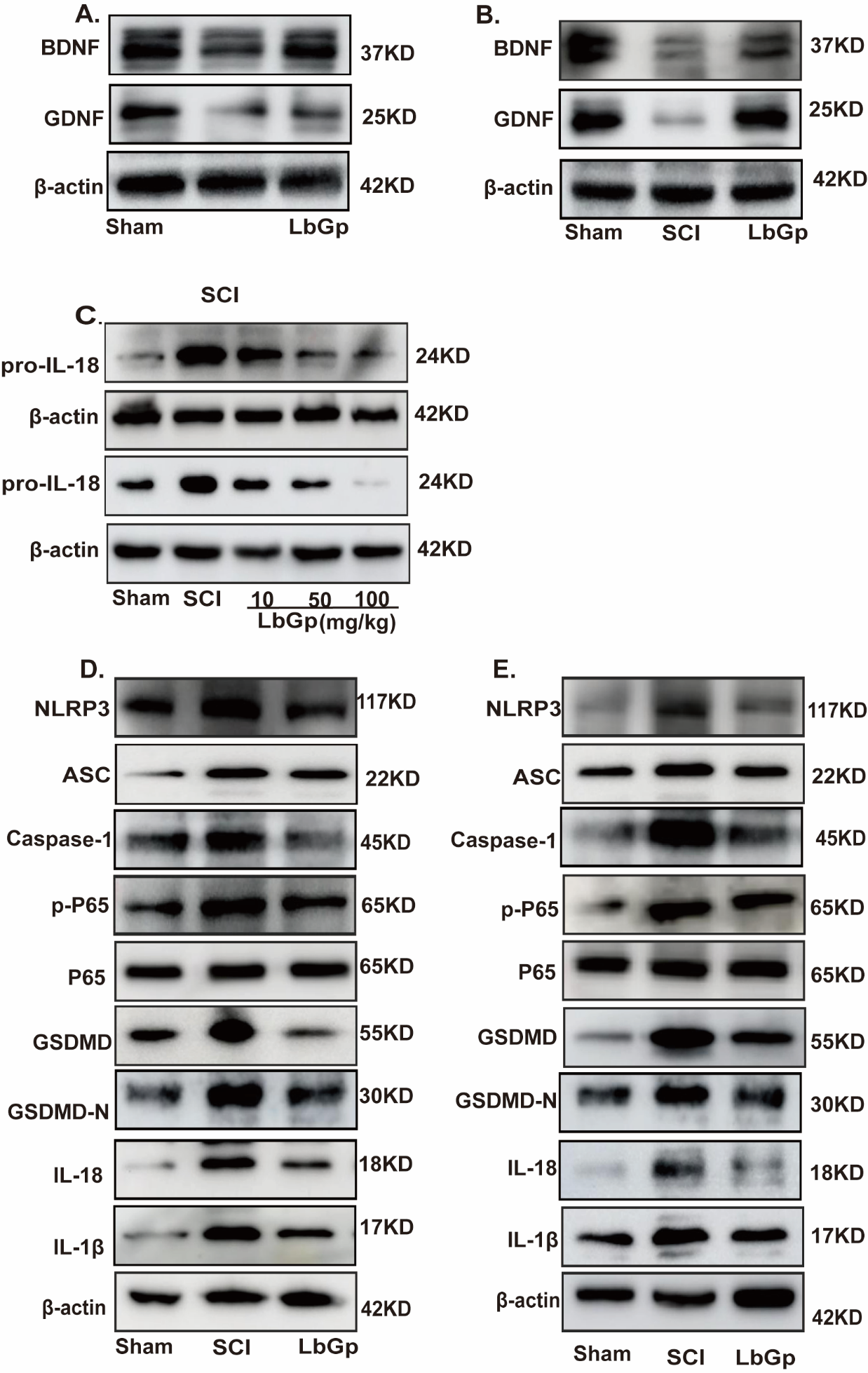
**

**Fig. S3 Effect of Lycium barbarum glycopeptide treatment on the spinal cord injury.** (A-B). detected the expression level of BDNF and GDNF in spinal cord tissue by western blotting. (C). Spinal cord injury treatment with different doses(10mg/kg-100mg/kg) of LbGp of reduces pro-IL-18 protein expression levels. （D-E）On day 7, the expression level of each group of NLRP3, ASC, caspase-1, P-p65, P65, GSDMD, GSDMD-N, IL-18, IL-1β protein in spinal cord tissue was detected by western blotting.


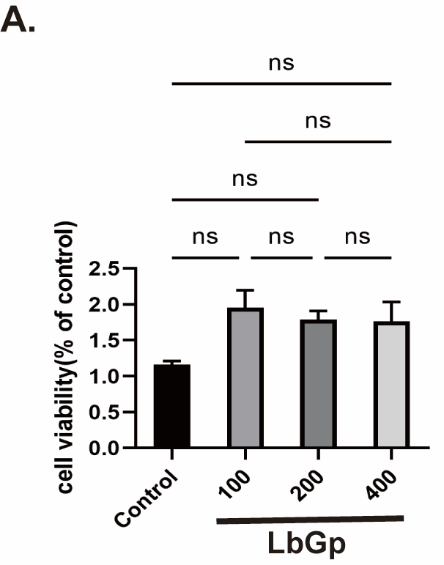


**Fig. S4 Cell viability of microglia treated with different concentration of Lycium barbarum glycopeptide.** (A)Cell viability of microglia treated with different concentration of LbGp

**
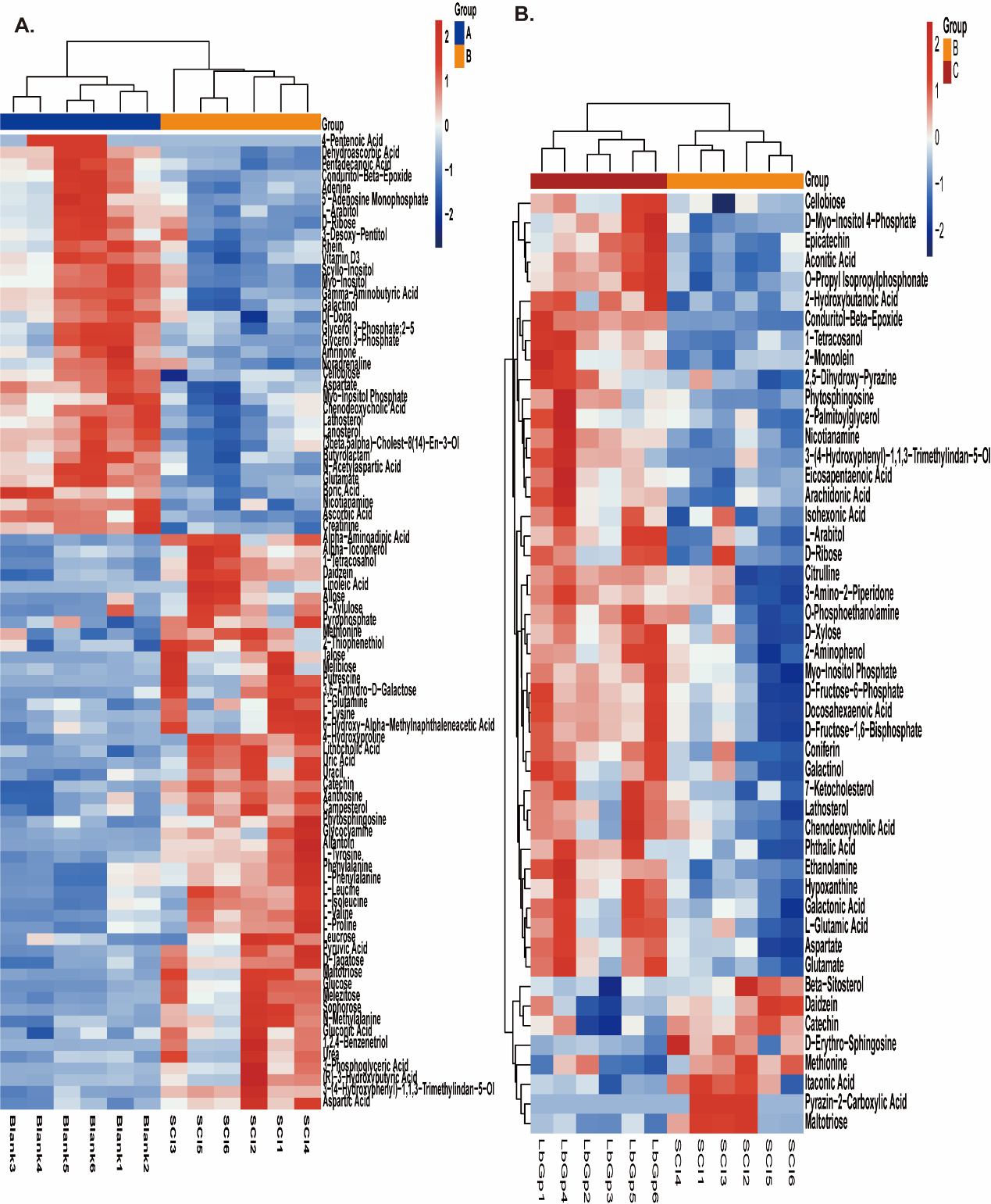
**


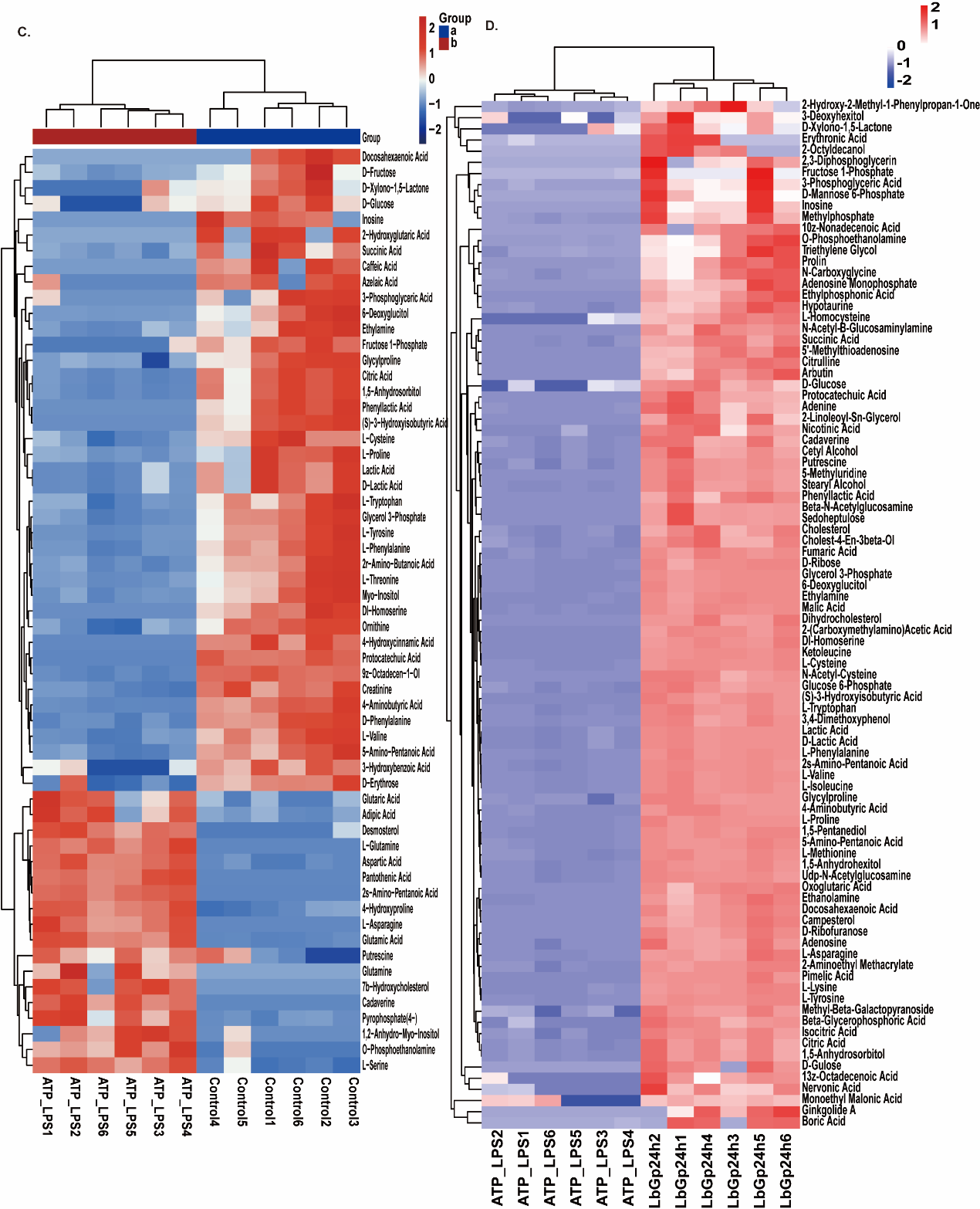


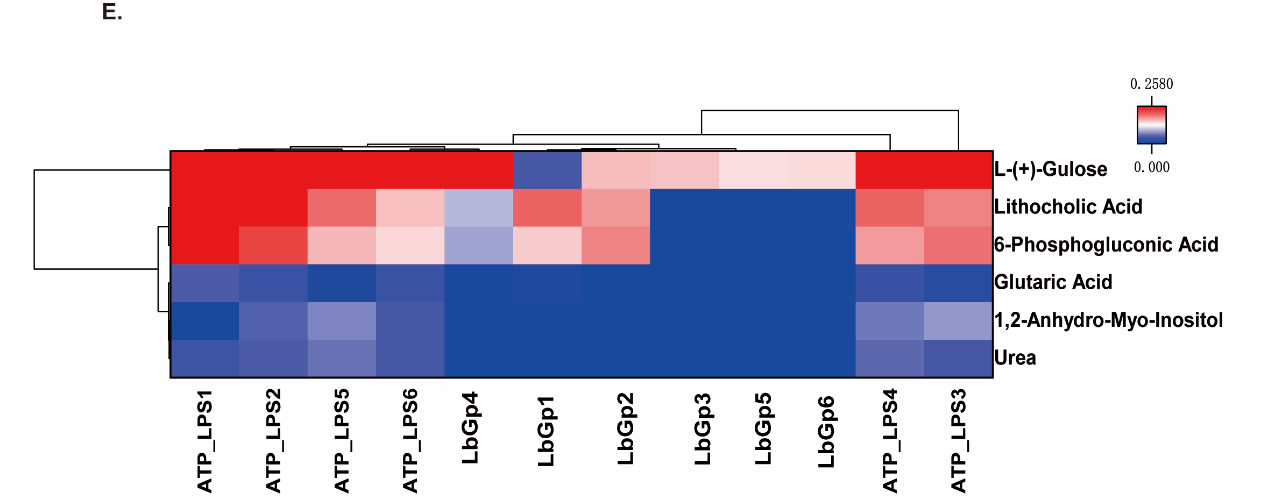


**Fig. S5 Heatmap of differential metabolic substances between three groups in vivo and vitro.** (A)Heatmap of differential metabolic substances between the Sham and LbGp groups in spinal cord tissue. (B)Heatmap of differential metabolic substances between the SCI and LbGp groups in spinal cord tissue. (C)Heatmap of differential metabolic substances between Control and ATP+LPS treatment groups in microglia supernatants. (D-E) Heatmap of differential metabolic substances between ATP+LPS and LbGp treatment groups in microglia supernatants.
