# Supplementary material for: Lycium barbarum glycopeptide alleviates neuroinflammation in spinal cord injury via modulating docosahexaenoic acid to inhibiting MAPKs/NF-kB and pyroptosis pathways
Source: J Transl Med. 2023 Oct 31;21:770. doi: 10.1186/s12967-023-04648-9 (PMC10617163; doi:10.1186/s12967-023-04648-9)
Supplement: Supplementary file 2 — Additional file 2: Table S1. Gene specific primer sequences of IL-18, FADS1 and FADS2. [file 12967_2023_4648_MOESM2_ESM.docx]

**Table S1. Gene specific primer sequences of IL-18, FADS1 and FADS2.**

1.Gene specific primer sequences used in the study.

| mRNA | Forward primer (5’-3’) | Forward primer (5’-3’) |
| --- | --- | --- |
| IL-18 | TGATATCGACCGAACAGCCAACG | GGTCACAGCCAGTCCTCTTACTTC |
| FADS1 | CCCACCAAGAATAAGGCGC | GTGAAGGGCACCAAGGAAGTT |
| FADS2 | GGAACCATCGACATTTCCAG | TCTTTATGTCGGGGTCCTTG |
| β-actin | ACTGCCGCATCCTCTTCCTC | CTCCTGCTTGCTGATCCACATC |
